# Enhanced Renoprotective Effects of Morin-Loaded PLGA Nanoparticles Against Arsenic-Induced Kidney Injury in Rats: Amelioration of Oxidative Stress, Inflammation, Fibrosis, and Apoptosis

**DOI:** 10.3390/ph19060871

**Published:** 2026-05-30

**Authors:** Abdulrahman S. Aldaghmi, Ekramy M. Elmorsy, Fahad Alshammari, Amro Duhduh, Nagwa M. Aly, Ola A. Habotta, Manal S. Fawzy, Shaimaa A. Shehata

**Affiliations:** 1 Department of Biology, College of Science, Jouf University, Sakaka 72341, Saudi Arabia; asaldaghmi@ju.edu.sa (A.S.A.); fahadm@ju.edu.sa (F.A.); 2 Center for Health Research, Northern Border University, Arar 73213, Saudi Arabia; ekramy.elmorsy@nbu.edu.sa; 3 Department of Medical Laboratory Technology, College of Nursing and Health Science, Jazan University, Jazan 45142, Saudi Arabia; aduhduh@jazanu.edu.sa; 4 Department of Medical Biochemistry and Molecular Biology, Faculty of Medicine, Suez Canal University, Ismailia 41522, Egypt; nagwaaly@med.suez.edu.eg; 5 Department of Forensic Medicine and Clinical Toxicology, Faculty of Veterinary Medicine, Mansoura University, Mansoura 35516, Egypt; ola_ali@mans.edu.eg; 6 Horus Research Center, Horus University—Egypt (HUE), New Damietta 34518, Egypt; 7 Department of Forensic Medicine and Clinical Toxicology, Faculty of Medicine, Suez Canal University, Ismailia 41522, Egypt; shaimaa_shehata@med.suez.edu.eg

**Keywords:** morin, PLGA nanoparticles, arsenic-induced nephrotoxicity, mining-related arsenic exposure, oxidative stress, inflammation, fibrosis, apoptosis, Nrf2/HO-1 pathway, TLR4/NF-κB signaling

## Abstract

**Background/Objectives**: Arsenic (ARS) exposure is a major cause of kidney injury, driven by oxidative stress, inflammation, fibrosis, and apoptosis. This study evaluated the renoprotective effects of morin (MOR) and morin-loaded PLGA nanoparticles (MOR–PGNPs) against ARS-induced nephrotoxicity in rats. **Methods**: Sixty male Sprague Dawley rats were randomly allocated into six groups (n = 10 per group). The control group received corn oil. The MOR group received MOR (100 mg/kg), and the MOR–PGNPs group received the same dose of MOR encapsulated in PLGA nanoparticles. ARS was administered at 10 mg/kg for 14 days. Co-treated groups received ARS together with either MOR or MOR–PGNPs, with a 28 min interval between administrations. Renal function markers (serum urea, creatinine, uric acid, renal KIM-1), oxidative stress and antioxidant parameters (Nrf2/HO-1, CAT, SOD, GPx, ROS, MDA), inflammatory mediators (TLR4/NF-κB, TNF-α, IL-6, IL-1β), fibrotic markers (TGF-β1, fibronectin), and apoptotic proteins (caspase-3, caspase-8, Bax, Bcl-2) were assessed, alongside histopathological and ultrastructural evaluations. **Results**: ARS exposure significantly impaired renal function, increased KIM-1, suppressed Nrf2/HO-1 signaling, reduced antioxidant enzyme activities, and elevated ROS and MDA levels. It also activated TLR4/NF-κB signaling, upregulated pro-inflammatory cytokines and fibrotic markers, and increased pro-apoptotic proteins while downregulating Bcl-2. MOR co-treatment partially ameliorated these alterations. MOR–PGNPs produced potentially enhanced protection, restoring kidney function markers, enhancing antioxidant defenses, and markedly attenuating inflammation, fibrosis, and apoptosis. Histopathological and ultrastructural analyses confirmed preservation of glomerular and tubular architecture, mitochondrial integrity, and minimal cytoplasmic vacuolization in the MOR–PGNPs group. **Conclusions**: MOR–PGNPs at 100 mg/kg effectively mitigated ARS-induced renal damage through antioxidant, anti-inflammatory, antifibrotic, and anti-apoptotic mechanisms, supporting PLGA-based morin nanoparticles as a promising and safe renoprotective strategy.

## 1. Introduction

Arsenic (ARS), a metalloid naturally found throughout the Earth’s crust, poses a significant environmental and public health challenge globally [1]. It exists primarily as arsenite (As^3+^) and arsenate (As^5+^), with arsenite being more toxic and more frequently detected in groundwater sources [2]. Human exposure to ARS occurs mainly through contaminated drinking water, ingestion of food grown in arsenic-contaminated soils, and inhalation of arsenic-laden dust [3]. ARS enters the environment through natural phenomena, including rock weathering and volcanic eruptions, as well as through human activities such as mining, fossil fuel combustion, improper chemical disposal, and pesticide use [4,5].

Following absorption, ARS accumulates in several tissues, with the kidneys being particularly vulnerable due to their critical detoxifying and excretory functions [6]. In renal tissues, ARS induces oxidative stress by increasing reactive oxygen species (ROS) production, leading to DNA damage, lipid peroxidation, and protein oxidation [4,7]. This oxidative insult is frequently accompanied by a pronounced inflammatory response, characterized by elevated levels of pro-inflammatory cytokines that further amplify tissue injury [8]. Chronic exposure to ARS can also contribute to renal fibrosis by promoting extracellular matrix deposition [9]. In addition, it disrupts apoptotic signaling, leading to increased cell death and progressive impairment of kidney function [4,10]. Together, these complex effects underscore the urgent need for strategies that can protect critical organs, particularly the kidneys, from ARS-induced damage.

Morin (MOR; 3,5,7,2′,4′-pentahydroxyflavone) is a naturally occurring bioflavonoid present in various fruits and medicinal herbs and is known for its antioxidant, anti-inflammatory, chemoprotective, and anticancer properties [11]. Its plant-derived antioxidant activity makes it a safer alternative to standard metal chelators for mitigating ARS toxicity [12]. Experimental studies have demonstrated that MOR can neutralize ROS, inhibit the production of inflammatory mediators, and regulate apoptotic pathways, indicating its capacity to protect kidney tissues from toxin-induced damage [13,14]. Despite this promising therapeutic profile, the clinical use of MOR is constrained by its extremely low oral bioavailability (<1%) and poor water solubility [15]. It also undergoes rapid hepatic metabolism and has a short circulating half-life of approximately 30 min [16]. These pharmacokinetic limitations compromise MOR’s tissue targeting and reduce its therapeutic efficacy, necessitating the development of innovative drug-delivery strategies.

Nanotechnology-enabled drug delivery has proven effective in overcoming the pharmacokinetic shortcomings of many natural compounds. Polymeric nanoparticles fabricated from poly(lactic-co-glycolic acid) (PLGA) are particularly attractive owing to their biodegradability and biocompatibility [17]. PLGA nanoparticles were specifically selected in this study because they are FDA-approved, exhibit an established safety profile, and provide efficient encapsulation, controlled release, and enhance the bioavailability of poorly soluble phytochemicals such as MOR. PLGA can be produced either by direct condensation of glycolic acid and lactic acid or through the ring-opening polymerization (ROP) of lactide and glycolide cyclic dimers [18]. Various initiators, including stannous octoate, tin(II) bis(2-ethylhexanoate) (Sn(Oct)_2_), and zinc proline, are commonly used in this process to generate PLGA copolymers with defined molecular weights and functional carboxyl and hydroxyl terminal groups [17,19].

In therapeutic applications, PLGA-based nanoparticles have demonstrated a remarkable capacity to enhance the stability, solubility, and bioavailability of bioactive compounds such as MOR [16]. Encapsulation of MOR in PLGA nanoparticles protects it from rapid metabolism and clearance, prolongs its systemic circulation, and promotes greater accumulation in target tissues, including the kidneys [17,20]. Additionally, PLGA nanoparticles provide controlled and sustained drug release [21], thereby maintaining effective therapeutic levels for longer periods while reducing the frequency of administration [22]. Collectively, these advantages may improve the body-wide distribution of MOR, potentiate its therapeutic actions, and minimize off-target side effects in non-target tissues. Consequently, PLGA nanoparticles represent a promising delivery system for fully exploiting the protective and therapeutic potential of natural bioflavonoids such as MOR in combating ARS-induced renal injury.

Accordingly, this study was designed to investigate the renoprotective effects of MOR-loaded PLGA nanoparticles (MOR–PGNPs) in a rat model of ARS-induced kidney injury. To the best of our knowledge, this is the first study to comprehensively evaluate the potential enhanced renoprotective efficacy of MOR–PLGA nanoparticles against ARS-induced nephrotoxicity. Unlike previous studies employing crude morin or other nanoformulated flavonoids, such as quercetin-, naringenin-, and curcumin-based nanoparticles, which have been evaluated primarily in cisplatin- or gentamicin-induced nephrotoxicity models and focused on limited mechanistic endpoints, the present work simultaneously interrogates four intertwined pathological axes: oxidative stress (Nrf2/HO-1 pathway), TLR4/NF-κB-mediated inflammation, TGF-β1-driven fibrosis, and caspase-dependent apoptosis, at both protein and gene expression levels. Furthermore, by maintaining the same nominal morin dose (100 mg/kg) across free and nanoencapsulated groups, this study specifically isolates the contribution of PLGA-based nanodelivery to the observed renoprotective enhancement. The study aims to determine whether this nanoformulation can more effectively mitigate oxidative stress, inflammation, fibrosis, and apoptosis in renal tissues compared to crude MOR. This approach highlights the potential of nanotechnology-based delivery of natural compounds to enhance therapeutic efficacy against ARS-induced nephrotoxicity.

## 2. Results

### 2.1. Physicochemical Characterization of MOR–PGNPs

**Figure 1:**
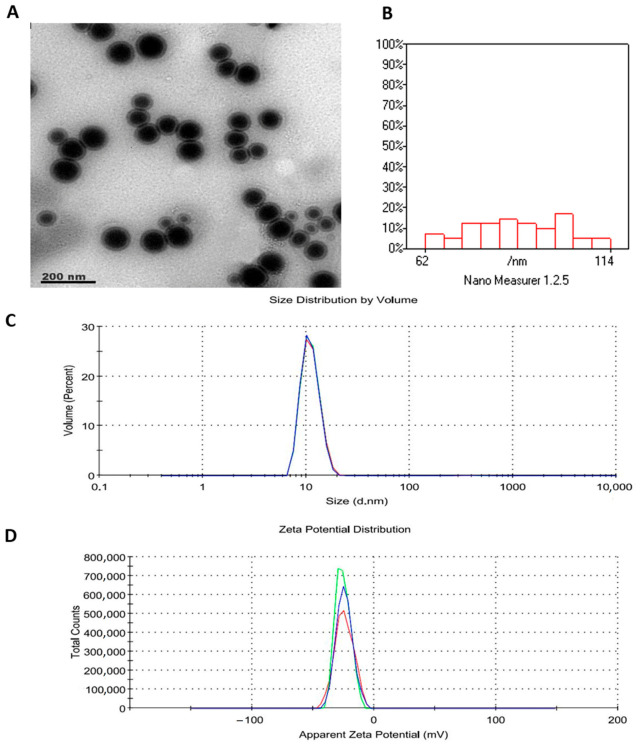
Morphology, size distribution, and surface charge of Morin-loaded PLGA nanoparticles. (**A**) Transmission electron microscopy (TEM) image showing predominantly spherical nanoparticles. (**B**) Histogram of size distribution indicating that most nanoparticles range from 62 to 114 nm. (**C**) Particle size distribution by volume. The blue line represents the volume-weighted size distribution curve generated by dynamic light scattering, while the overlaid red line indicates the fitted distribution used by the instrument software to calculate the mean hydrodynamic diameter and polydispersity index. (**D**) Zeta potential distribution. The green line corresponds to the main zeta potential distribution of the nanoparticle suspension; the red line shows the fitted peak used to derive the mean zeta potential; and the blue line represents the overall distribution envelope obtained from the electrophoretic light scattering measurement. The formulation exhibited a Z-average size of 118 nm, a polydispersity index (PDI) of 0.403, and a zeta potential of −24 mV, indicating a well-dispersed and colloidally stable nanoparticle population.

The MOR–PGNPs appeared as discrete, spherical vesicles with preserved structural integrity and no visible aggregation (Figure 1A). Particle size analysis revealed a size range of 62–114 nm (Figure 1B). Dynamic light scattering measurements yielded an average hydrodynamic diameter of 118 nm and a polydispersity index (PDI) of 0.403 (Figure 1C), reflecting a moderately polydisperse population within the acceptable range reported for PLGA nanoparticles encapsulating poorly water-soluble phytochemicals prepared by nanoprecipitation/emulsification methods. The MOR–PGNPs exhibited a zeta potential of −24 mV (Figure 1D), suggesting a negatively charged surface and good colloidal stability of the nanoparticle suspension.

### 2.2. Effect of Storage on the Physical Stability of MOR–PGNPs

Table 1 shows that MOR–PGNPs maintained acceptable physical stability during storage at 4 °C for 14 days, with only minor, non-significant changes in particle size, PDI, and zeta potential. Particle size increased marginally from 118 to 125 nm over the 14 days, accompanied by a slight rise in PDI, indicating a minor but acceptable shift in size distribution. The zeta potential decreased slightly from −24.0 to −21.2 mV, while still reflecting sufficient surface charge to maintain colloidal stability and prevent aggregation. Overall, the formulation remained physically stable under the tested storage conditions throughout the experimental period.

### 2.3. Entrapment Efficiency (EE) and Drug Loading (DL)

Morin–PLGA nanoparticles exhibited an entrapment efficiency of approximately 82.17% and a drug loading capacity of approximately 16.13%, indicating efficient morin incorporation into the PLGA matrix and confirming the suitability of the preparation method for sustained-drug-delivery applications.

### 2.4. Fourier Transform Infrared (FTIR) Analysis Results

**Figure 2:**
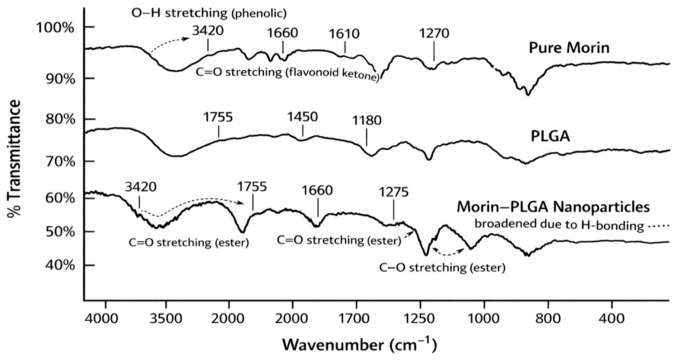
FTIR spectra of pure morin, PLGA, and morin–PLGA nanoparticles; Characteristic peaks for morin (–OH, C=O, C=C, C–O–C) and PLGA (ester C=O, C–O–C, CH_3_) are shown. The broadening of the –OH peak and a slight shift in the C=O peak in the nanoparticles confirm the successful encapsulation of morin within PLGA.

The FTIR spectra of pure morin, PLGA, and morin–PLGA nanoparticles showed distinct peaks corresponding to their characteristic functional groups. For pure morin, a broad band at 3420 cm^−1^ indicated phenolic –OH stretching, while peaks at 1660 cm^−1^ and 1610 cm^−1^ were attributed to C=O stretching of the flavonoid ketone and aromatic C=C stretching, respectively. The C–O–C stretching vibration appeared at 1270 cm^−1^. The spectrum of PLGA displayed a strong ester carbonyl peak at 1755 cm^−1^, C–O–C stretching at 1180 cm^−1^, and CH_3_ bending at 1450 cm^−1^, confirming the polymer’s characteristic ester linkages. In the morin–PLGA nanoparticles, the –OH band at 3420 cm^−1^ became broader, reflecting hydrogen-bonding interactions between morin and the polymer. The ester C=O peak of PLGA remained at 1755 cm^−1^, whereas the morin C=O band at 1660 cm^−1^ showed a slight shift, suggesting weak non-covalent interactions with PLGA without chemical degradation. Overall, the FTIR data support the successful encapsulation of morin within PLGA nanoparticles without altering the fundamental chemical structures of either component (Figure 2).

### 2.5. In Vitro Drug Release

**Figure 3:**
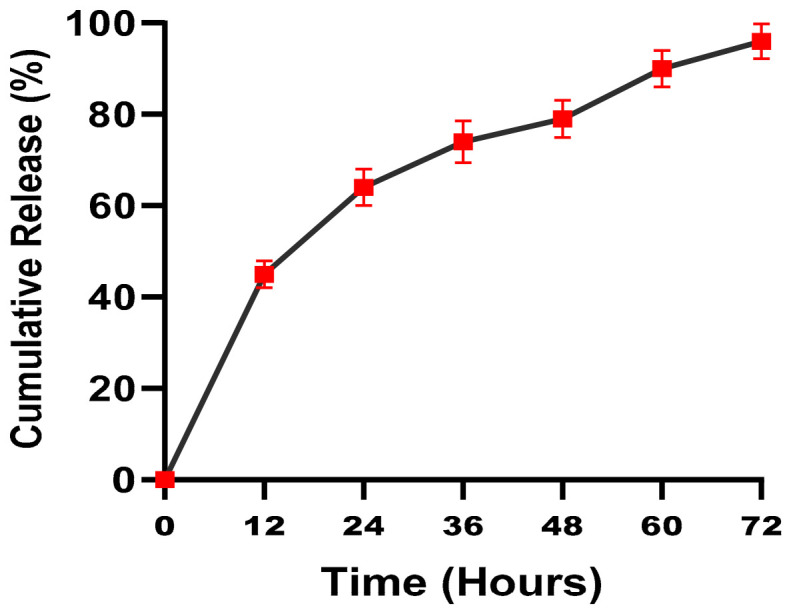
In vitro release of morin from PLGA nanoparticles over 72 h, showing sustained and controlled drug release, reaching nearly 96% by the end of the study.

Figure 3 illustrates the in vitro release profile of morin from PLGA nanoparticles over 72 h. The release pattern was sustained, with an initial moderate release during the first 12 h, followed by a gradual increase over time. By 48 h, approximately 80% of the encapsulated morin had been released, and the release reached near-complete levels (~96%) by 72 h. This gradual, controlled release profile indicates that the PLGA nanoparticles effectively modulate morin delivery, providing extended drug availability and minimizing burst release.

### 2.6. In Vitro Release Kinetic Modeling Results

The in vitro release data were fitted to zero-order, first-order, Higuchi, and Korsmeyer–Peppas kinetic models to elucidate the predominant release mechanism. The Higuchi model provided the best fit, with the highest correlation coefficient (R^2^ = 0.986), followed by the Korsmeyer–Peppas model (R^2^ = 0.970), collectively indicating that diffusion-controlled release was the predominant mechanism governing morin liberation from the PLGA matrix. The Korsmeyer–Peppas release exponent (n = 0.57) was consistent with a non-Fickian (anomalous) transport mechanism, involving a combination of drug diffusion and polymer matrix relaxation/erosion. Lower correlation coefficients were observed for zero-order (R^2^ = 0.922) and first-order kinetics (R^2^ = 0.892), confirming that morin release did not follow strictly constant or concentration-dependent kinetics.

### 2.7. Effect of Morin and MOR–PLGA Nanoparticles on Kidney Function Markers

**Figure 4:**
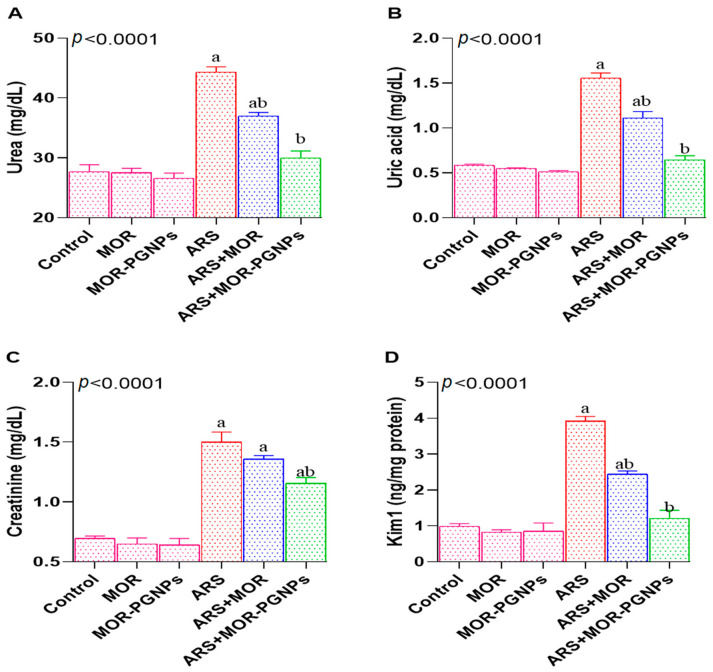
Renal function markers in arsenic-exposed rats following co-treatment with morin and MOR–PLGA nanoparticles. Sub-acute arsenic administration produced marked elevations in serum urea (**A**), uric acid (**B**), and creatinine (**C**), alongside a significant increase in renal kidney injury molecule-1 (KIM-1; (**D**)), relative to the normal control group. Co-treatment with MOR and MOR–PGNPs progressively attenuated these alterations, with the nanoparticle formulation demonstrating better restoration of all markers. Values are expressed as mean ± SE (n = 7). Bars carrying different superscript letters (“a”, “b”) differ significantly at *p* < 0.05 from the normal control (“a”) or ARS-treated group (“b”), respectively.

Exposure to ARS significantly impaired kidney function, as evidenced by elevated serum levels of urea (Figure 4A), uric acid (Figure 4B), and creatinine (Figure 4C), along with increased renal KIM-1 levels (Figure 4D), compared with the normal control group. Co-treatment with the PLGA-loaded form of MOR significantly reduced serum urea, creatinine, and uric acid levels; urea and uric acid were restored to values that did not differ significantly from those of the normal control group. In contrast, no significant difference in creatinine levels was observed between the ARS-only group and the ARS + crude MOR group.

In KIM-1, treatment with both crude MOR and MOR–PGNPs, combined with ARS, significantly reduced renal levels compared with the ARS-treated group, with the lowest values observed in the MOR–PGNPs-treated group. In this group, KIM-1 levels were restored to values that did not differ significantly from those of the normal control group (Figure 4).

### 2.8. Effect of Morin and MOR–PLGA Nanoparticles on Nrf2/HO-1 and Oxidative Stress

**Figure 5:**
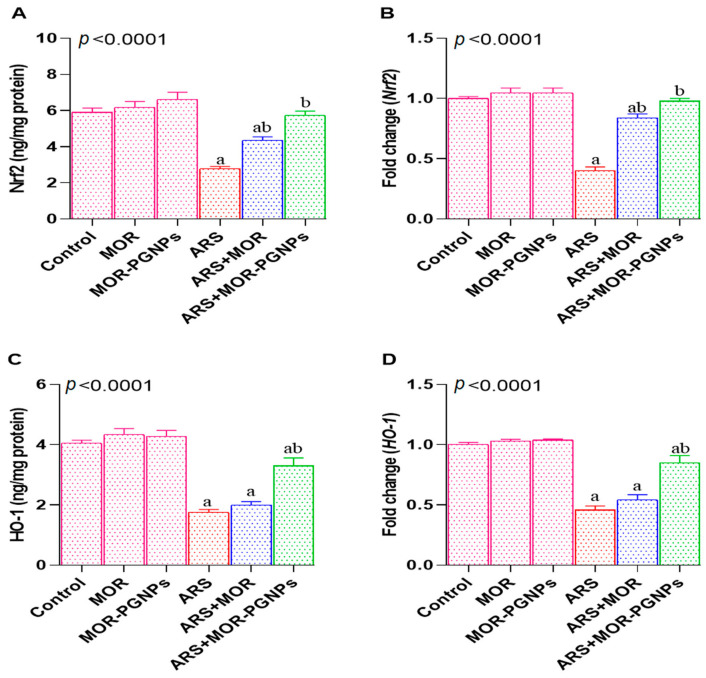
Renal Nrf2/HO-1 antioxidant signaling in arsenic-exposed rats treated with morin and MOR–PLGA nanoparticles. Sub-acute arsenic administration markedly suppressed the expression of nuclear factor erythroid 2-related factor 2 (Nrf2) and its downstream cytoprotective target heme oxygenase-1 (HO-1) in renal tissues. Panels (**A**) and (**B**) depict Nrf2 protein and mRNA expression levels, respectively, while panels (**C**) and (**D**) illustrate the corresponding protein and mRNA expression of HO-1. Values are expressed as mean ± SE (n = 7). Bars carrying different superscript letters (“a”, “b”) differ significantly at *p* < 0.05 from the normal control (“a”) or ARS-treated group (“b”), respectively.

Exposure to ARS markedly suppressed the Nrf2/HO-1 signaling pathway, as indicated by significant reductions in Nrf2 (Figure 5A,B) and its downstream target HO-1 (Figure 5C,D) at both protein and mRNA levels compared with the normal control group. Co-treatment with MOR in both forms significantly increased Nrf2 expression relative to the ARS-treated group, with a more pronounced effect in the MOR–PGNPs group, where Nrf2 levels did not differ significantly from those of the normal control group.

Similarly, administration of PLGA-loaded MOR significantly increased HO-1 expression at both the protein and transcriptional levels compared with the ARS-treated group. In contrast, no significant difference in HO-1 levels was detected between the ARS-treated group and the ARS + crude MOR group.

**Figure 6:**
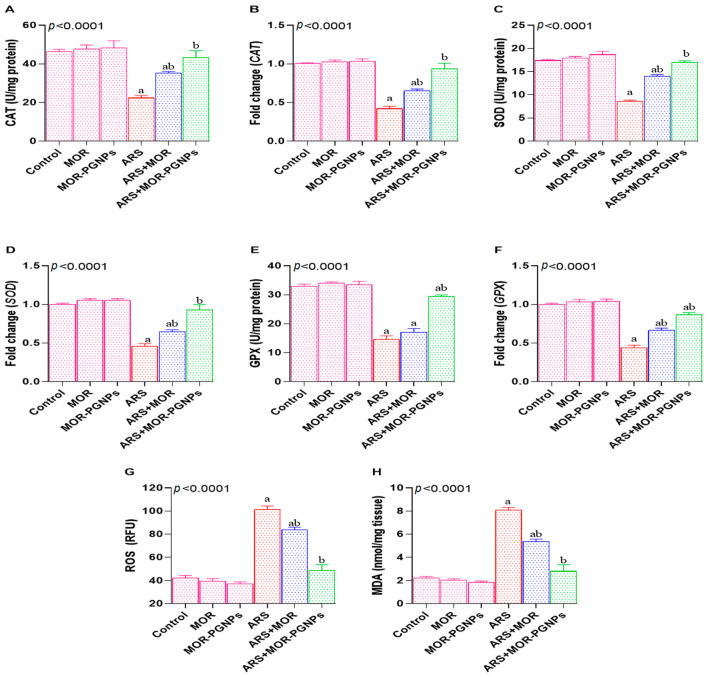
Renal antioxidant enzyme activities and oxidative stress biomarkers following arsenic exposure and morin-based interventions. Panels (**A**,**B**) depict catalase (CAT) protein and transcript expression, respectively; panels (**C**,**D**) present superoxide dismutase (SOD) at protein and transcriptional levels; panels (**E**,**F**) illustrate glutathione peroxidase (GPx) protein and gene expression. Panel (**G**) reflects intracellular reactive oxygen species (ROS) accumulation, and panel (**H**) represents malondialdehyde (MDA) content as an index of lipid peroxidation. Values are expressed as mean ± SE (n = 7). Bars carrying different superscript letters (“a”, “b”) differ significantly at *p* < 0.05 from the normal control (“a”) or ARS-treated group (“b”), respectively.

In parallel, the activities and expression levels of antioxidant enzymes, including CAT (Figure 6A,B), SOD (Figure 6C,D), and GPx (Figure 6E,F), were significantly reduced after ARS exposure at both protein and gene expression levels compared with the normal control group. Co-administration of MOR in both forms with ARS significantly enhanced these antioxidant parameters relative to the ARS-treated group. Notably, the nanoparticle formulation restored CAT and SOD to levels that did not differ significantly from those of the normal control group. Treatment with MOR–PGNPs also significantly improved GPx activity compared with the ARS-only group, whereas no significant difference was observed between the ARS-treated and ARS + crude MOR groups in GPx protein levels.

Regarding oxidative stress biomarkers, ARS exposure significantly increased renal ROS (Figure 6G) and lipid peroxidation, as indicated by MDA levels (Figure 6H), compared with the normal control group. Co-treatment with both forms of MOR significantly reduced ROS and MDA levels compared with the ARS-treated group. The lowest values were recorded in the ARS + MOR–PGNPs group, in which ROS and MDA did not differ significantly from those in the normal control group (Figure 6).

### 2.9. Effect of Morin and MOR–PLGA Nanoparticles on TLR4/NF-κB Pathway and Inflammatory Cytokines

**Figure 7:**
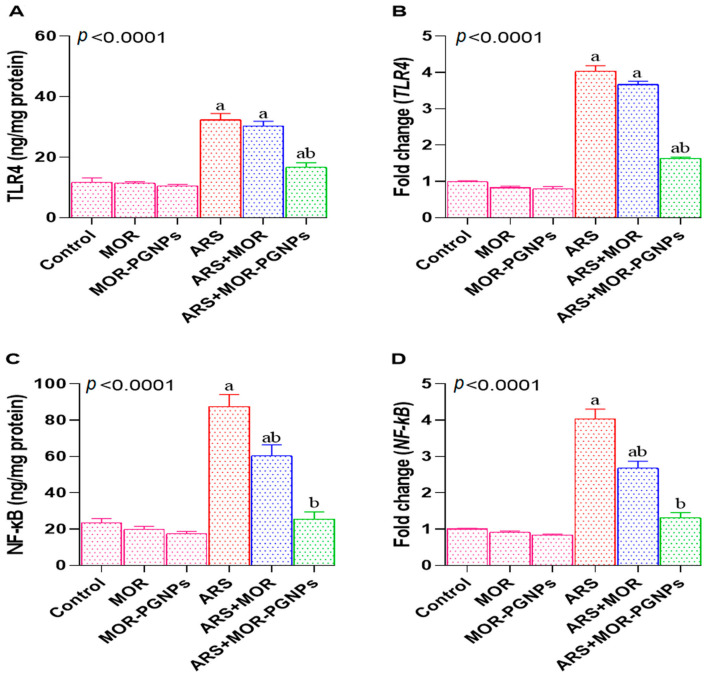
Renal TLR4/NF-κB inflammatory signaling in response to arsenic exposure and morin-based treatments. Sub-acute arsenic administration markedly upregulated the expression of Toll-like receptor 4 (TLR4) and nuclear factor kappa-B (NF-κB) in renal tissues relative to the normal control group. Panels (**A**,**B**) represent TLR4 protein and transcript levels, respectively; panels (**C**,**D**) depict the corresponding protein and transcript of NF-κB. Values are expressed as mean ± SE (n = 7). Bars sharing different superscript letters (“a”, “b”) are significantly different at *p* < 0.05 from the control group (“a”) or the ARS-treated group (“b”), respectively.

Both TLR4 and NF-κB showed significant upregulation at protein and mRNA levels in ARS-exposed rats relative to the normal control group (Figure 7A–D). When MOR was co-administered with ARS, a meaningful reduction in TLR4 and NF-κB expression was observed, although this effect was considerably more pronounced in animals receiving MOR–PGNPs. Notably, NF-κB protein and gene expression in the MOR–PGNPs-treated group returned to values statistically comparable to those of the normal control, while TLR4 suppression in this group surpassed that achieved by crude MOR. In contrast, TLR4 expression remained statistically indistinguishable between the ARS-only and ARS + crude MOR groups.

**Figure 8:**
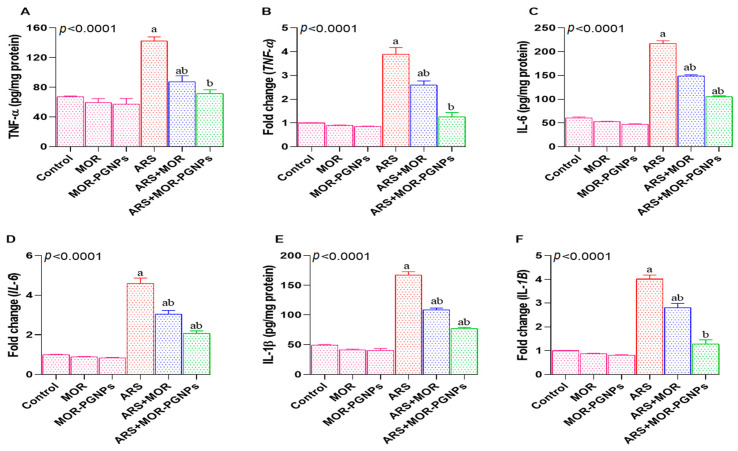
Renal pro-inflammatory cytokine expression in arsenic-exposed rats following co-treatment with morin and MOR–PLGA nanoparticles. Sub-acute arsenic administration drove marked upregulation of tumor necrosis factor-alpha (TNF-α; panels (**A**,**B**)), interleukin-6 (IL-6; panels (**C**,**D**)), and interleukin-1 beta (IL-1β; panels (**E**,**F**)) at both protein and transcriptional levels in renal tissues relative to the normal control group. Co-treatment with MOR partially suppressed these cytokines, whereas MOR–PGNPs produced a substantially greater anti-inflammatory effect across all three markers. Values are expressed as mean ± SE (n = 7). Bars carrying different superscript letters (“a”, “b”) differ significantly at *p* < 0.05 from the normal control (“a”) or ARS-treated group (“b”), respectively.

Consistent with these findings, ARS exposure significantly elevated the levels of pro-inflammatory cytokines in renal tissues. TNF-α (Figure 8A,B), IL-6 (Figure 8C,D), and IL-1β (Figure 8E,F) were markedly increased at both protein and mRNA levels compared with the normal control group. Co-treatment with MOR in both forms significantly reduced these cytokines compared with the ARS-treated group. The nanoparticle formulation exerted a more pronounced anti-inflammatory effect than crude MOR, with no significant differences between the ARS + MOR–PGNPs and normal control groups for TNF-α protein and mRNA levels, or for IL-1β mRNA expression (Figure 7 and Figure 8).

### 2.10. Effect of Morin and MOR–PLGA Nanoparticles on Fibrotic Markers

**Figure 9:**
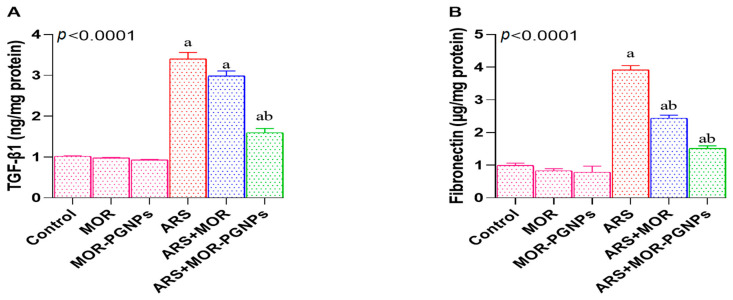
Renal fibrosis markers in arsenic-exposed rats following co-treatment with morin and MOR–PLGA nanoparticles. Sub-acute arsenic administration significantly elevated renal levels of transforming growth factor-beta 1 (TGF-β1; panel (**A**)) and fibronectin (panel (**B**)) relative to the normal control group, reflecting activation of profibrotic signaling and extracellular matrix accumulation. Co-treatment with MOR partially attenuated these fibrotic changes, whereas MOR–PGNPs produced a marked reduction in both markers. Values are expressed as mean ± SE (n = 7). Bars carrying different superscript letters (“a”, “b”) differ significantly at *p* < 0.05 from the normal control (“a”) or ARS-treated group (“b”), respectively.

The renal levels of TGF-β1 (Figure 9A) and fibronectin (Figure 9B) were significantly elevated in the ARS-treated group compared with the normal control group, indicating induction of renal fibrosis. Co-administration of MOR with ARS reduced the levels of these fibrotic markers, with a more pronounced effect observed in the MOR–PLGA nanoparticle-treated group. In particular, the PLGA formulation significantly decreased TGF-β1 and fibronectin compared with the ARS-treated group and displayed a stronger antifibrotic effect than crude MOR. In contrast, the ARS + crude MOR group showed no significant difference from the ARS-only group in TGF-β1 levels (Figure 9).

### 2.11. Effect of Morin and MOR–PLGA Nanoparticles on Apoptotic Markers

**Figure 10:**
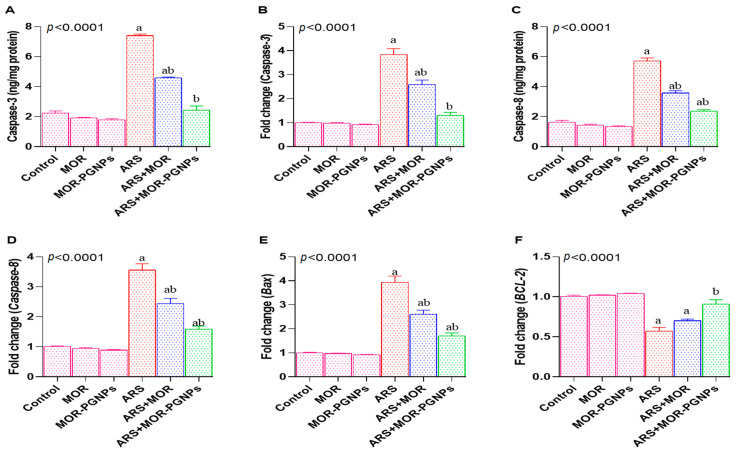
Renal apoptotic marker expression in arsenic-exposed rats following co-treatment with morin and MOR–PLGA nanoparticles. Sub-acute arsenic administration significantly dysregulated the apoptotic machinery in renal tissues, as evidenced by marked upregulation of caspase-3 (panels (**A**,**B**)), caspase-8 (panels (**C**,**D**)), and Bax (panel (**E**)) at protein and/or mRNA levels, alongside a concurrent downregulation of the anti-apoptotic protein Bcl-2 (panel (**F**)), relative to the normal control group. Co-treatment with MOR partially corrected this imbalance, whereas MOR–PGNPs produced a relatively complete restoration of pro- and anti-apoptotic marker levels. Values are expressed as mean ± SE (n = 7). Bars carrying different superscript letters (“a”, “b”) differ significantly at *p* < 0.05 from the normal control (“a”) or ARS-treated group (“b”), respectively.

Exposure to ARS significantly increased the levels of pro-apoptotic biomarkers in renal tissues. Caspase-3 and caspase-8 were elevated at both protein and mRNA levels (Figure 10A–D), and Bax mRNA expression was significantly increased compared with the normal control group (Figure 10E). Co-administration of MOR with ARS significantly reduced these pro-apoptotic markers, with the PLGA nanoparticle formulation showing a more pronounced anti-apoptotic effect than crude MOR. In the ARS + MOR–PGNPs group, caspase-3 protein and mRNA levels were restored to values that did not differ significantly from those of the normal control group (Figure 10A,B).

Conversely, the anti-apoptotic marker Bcl-2 was significantly decreased following ARS exposure compared with the negative control (Figure 10F). Treatment with PLGA-formulated MOR significantly restored Bcl-2 expression to levels comparable to the normal control group, whereas no significant difference was observed between the ARS + crude MOR and ARS-only groups (Figure 10F).

### 2.12. Effect of Morin and MOR–PLGA Nanoparticles on Kidney Histopathology

**Figure 11:**
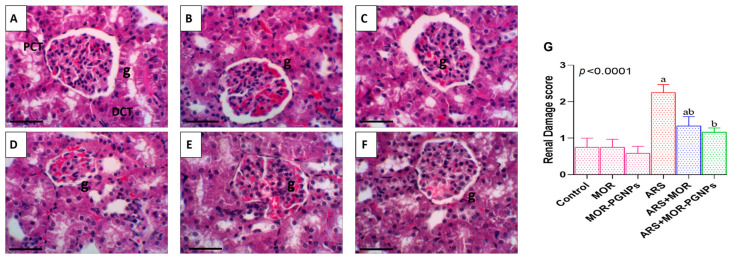
Representative photomicrographs of kidney tissues showing the impact of ARS and the protective effect of MOR. (**A**) Control group showing well-preserved cortical architecture with intact glomeruli (g), normally organized proximal convoluted tubules (PCT), and distal convoluted tubules (DCT). (**B**) The MOR-treated group displayed normal glomerular and tubular morphology comparable to the control. (**C**) The MOR–PGNPs-treated group maintained overall normal renal architecture.; (**D**) ARS-treated group exhibiting severe histopathological alterations, including vascular congestion, glomerular atrophy (g), and periglomerular lymphocytic infiltration. (**E**) ARS + MOR group showing partial amelioration of ARS-induced lesions with moderate preservation of glomerular and tubular structures. (**F**) ARS + MOR–PGNPs group demonstrated markedly improved renal architecture with well-preserved glomeruli (g) and tubular structures and minimal residual damage, indicating potentially enhanced renoprotection. All images captured at 400× magnification; scale bar = 50 µm. (**G**) Semi-quantitative renal histopathological damage scores across all experimental groups, confirming a significant increase in the ARS group and graded amelioration following MOR and MOR–PGNPs treatment, with MOR–PGNPs affording the greatest protection. Data are shown as mean ± SE. Different superscripts (“a”, “b”) indicate statistically significant differences (*p* < 0.05) from the normal control group (“a”) or the ARS-treated group (“b”).

Histological examination of renal tissues showed that the negative control group had well-preserved cortical and medullary architecture with normal glomeruli and tubules (Figure 11A). The MOR-treated group also displayed intact glomerular and tubular structures (Figure 11B), and the MOR–PGNPs group maintained an overall normal renal architecture (Figure 11C). In contrast, ARS exposure induced severe histopathological alterations, including vascular congestion, glomerular atrophy, and periglomerular lymphocytic infiltration (Figure 11D). Crude MOR treatment produced noticeable protection, with partial improvement of these lesions (Figure 11E). Remarkably, MOR–PGNPs treatment markedly alleviated vascular congestion and afforded better preservation of glomeruli and tubules compared with crude MOR (Figure 11F).

Consistent with these qualitative observations, renal damage scores were significantly higher in the ARS-exposed group than in the negative control. Crude MOR treatment led to moderate amelioration, whereas MOR–PGNPs provided the greatest protection, significantly lowering renal damage scores toward control values (Figure 11G).

### 2.13. Effect of Morin and MOR–PLGA Nanoparticles on Kidney Ultrastructure

**Figure 12:**
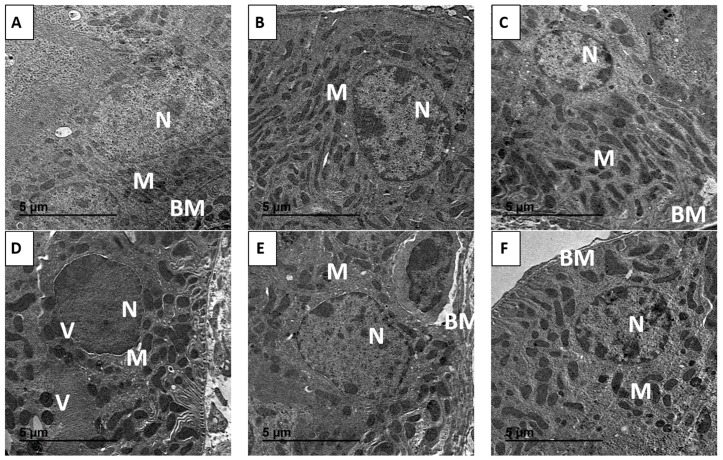
Electron micrographs of the renal cortex showing the effect of ARS exposure and the protective effect of MOR. (**A**) Control group; (**B**) MOR alone; (**C**) MOR–PGNPs; (**D**) ARS; (**E**) ARS + MOR; (**F**) ARS + MOR–PGNPs. Nucleus (N), mitochondria (M), Vacuole (V), and basement membrane (BM) are labeled.

As shown in Figure 12A–C, proximal tubular epithelial cells from the control, crude MOR, and MOR–PGNPs groups preserved normal ultrastructural features, including intact basement membranes, dense apical microvilli, elongated mitochondria, and rounded euchromatic nuclei. In contrast, proximal tubular cells from the ARS group exhibited marked injury, characterized by condensed, heterochromatic nuclei, extensive basal cytoplasmic vacuolization, and rounded, small, and structurally disrupted mitochondria, together with irregular and shortened apical microvilli (Figure 12D). Co-administration of MOR or MOR–PLGA markedly ameliorated these ultrastructural alterations. Proximal tubular cells in the treated groups showed largely restored mitochondrial morphology, rounded euchromatic nuclei, and dense apical microvilli, with only minimal residual cytoplasmic vacuolization compared with the ARS group (Figure 12E,F).

### 2.14. Assessment of Nrf2 Immunoreactivity

**Figure 13:**
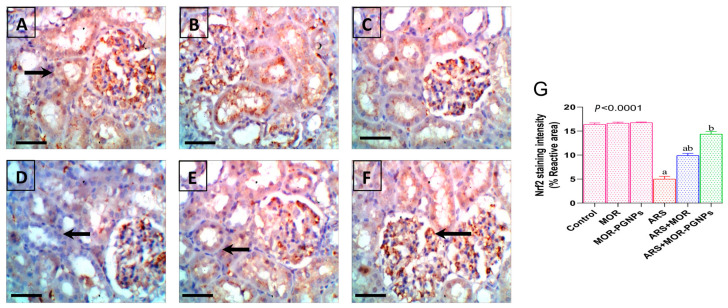
Representative photomicrographs showing NRF2 localization in rat renal tissue. (**A**–**C**) Control, MOR-only, and MOR–PGNPs-only show strong and widespread NRF2 immunoreactivity in cortical tubular epithelial cells, consistent with active antioxidant defense under basal conditions. (**D**) The ARS-treated group exhibits markedly reduced Nrf2 immunoreactivity, with only faint staining in tubular epithelial cells (black arrows), reflecting impaired antioxidant signaling. (**E**) The ARS + Morin group shows partial restoration of Nrf2 expression, with moderate staining in cortical tubules. (**F**) The ARS + MOR–PLGA nanoparticles group shows strong, widespread Nrf2 immunoreactivity throughout the cortex, approaching control levels. Original magnification ×400; scale bar = 50 µm. (**G**) Quantitative analysis of mean Nrf2 immunostaining intensity (% reactive area) across all experimental groups, confirming a significant reduction in the ARS group and graded restoration following MOR and MOR–PGNPs treatment. Data are presented as mean ± SE. Different superscripts (“a”, “b”) indicate statistically significant differences (*p* < 0.05) from the normal control group (“a”) or the ARS-treated group (“b”).

In renal sections from the control group, Nrf2 expression was pronounced, with tubular epithelial cells showing intense immunostaining and widespread Nrf2 activation across the cortical regions, consistent with an active antioxidant defense under basal conditions (Figure 13A–C). ARS exposure markedly reduced Nrf2 immunoreactivity, yielding faint staining in tubular epithelial cells and indicating impaired antioxidant protection (Figure 13D). Treatment with crude MOR partially restored Nrf2 expression, as reflected by moderate staining in cortical tubules (Figure 13F), whereas MOR–PGNPs induced strong and widespread Nrf2 immunoreactivity throughout the cortex, approaching or exceeding control levels (Figure 13E). These qualitative observations were confirmed by quantitative analysis of Nrf2 staining intensity, which showed a significant decrease in the ARS group and a more pronounced recovery in MOR–PGNPs-treated rats compared with crude MOR (Figure 13G).

### 2.15. Assessment of NF-κB Immunoreactivity

**Figure 14:**
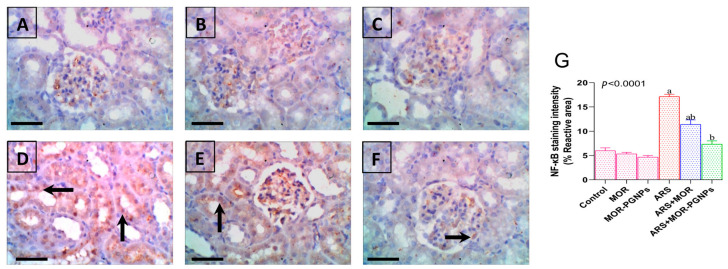
Representative photomicrographs showing NF-κB protein expression in rat renal tissue. (**A**–**C**) Control, MOR-only, and MOR–PGNPs-only groups exhibit minimal or absent NF-κB immunostaining in cortical tubular cells, consistent with a quiescent inflammatory state under basal conditions. (**D**) The ARS-treated group displays markedly enhanced NF-κB immunoreactivity, particularly in proximal tubular epithelial cells surrounding the glomeruli, as indicated by intense brown DAB staining (black arrows), reflecting activation of inflammatory signaling. (**E**) The ARS + Morin group shows a partial reduction in NF-κB expression, with moderate staining in cortical tubules (black arrow). (**F**) The ARS + MOR–PLGA nanoparticles group exhibits markedly diminished NF-κB immunoreactivity, with only faint staining approaching control levels (black arrow), indicating enhanced anti-inflammatory protection conferred by the nanoparticle formulation. Original magnification ×400; scale bar = 50 µm. (**G**) Quantitative analysis of mean NF-κB immunostaining intensity (% reactive area) across all experimental groups, confirming a significant increase in the ARS group and graded reduction following MOR and MOR–PGNPs treatment, with MOR–PGNPs achieving the greatest normalization. Data are presented as mean ± SE. Different superscripts (“a”, “b”) indicate statistically significant differences (*p* < 0.05) from the normal control group (“a”) or the ARS-treated group (“b”).

NF-κB expression was very low in control kidneys, with tubular cells showing almost no staining, indicating a quiescent inflammatory state under normal conditions (Figure 14A–C). Following ARS exposure, NF-κB immunoreactivity was markedly enhanced across the renal cortex (Figure 14D), reflecting activation of inflammatory signaling compared with controls. Treatment with MOR or MOR–PGNPs (Figure 14E,F) substantially reduced NF-κB expression, demonstrating a protective anti-inflammatory effect. Staining in cortical tubules was minimal and nearly returned to control levels in rats receiving MOR–PGNPs, indicating that the nanoparticle formulation was more effective than crude MOR in normalizing NF-κB activation (Figure 14G).

## 3. Discussion

Arsenic is a common environmental contaminant that poses a serious threat to human and animal health, with the kidney being particularly vulnerable due to its central role in detoxification and waste excretion [23]. ARS-induced kidney injury is now recognized as a multifactorial process involving oxidative stress, inflammation, and apoptosis, which together promote progressive renal fibrosis [4,9]. In the present study, we evaluated the protective effects of MOR and MOR–PGNPs against ARS-induced renal injury in rats. ARS exposure caused a significant decline in kidney function, evidenced by elevated serum creatinine, urea, and uric acid levels, which are established indicators of nephrotoxicity and impaired glomerular filtration [24]. Such renal dysfunction has been attributed to oxidative-stress-driven tubular epithelial injury and glomerular damage [6,9,25,26]. Notably, MOR administration, particularly in its nanoparticle-loaded form, substantially alleviated these alterations, significantly lowering serum creatinine, urea, and uric acid, likely through its potent antioxidant activity [14].

Histopathological examination supported the biochemical data. Kidneys from ARS-treated rats showed severe structural alterations, including tubular degeneration, inflammatory cell accumulation, glomerular atrophy, and epithelial cell necrosis. In contrast, MOR treatment preserved renal architecture, limited inflammatory infiltration, and reduced necrotic changes, demonstrating strong nephroprotective potential. Ultrastructural analysis further confirmed these findings, revealing that ARS induced mitochondrial swelling, disrupted cristae, nuclear condensation, and cytoplasmic vacuolization, whereas MOR, particularly MOR–PGNPs, markedly restored mitochondrial morphology and nuclear integrity. These observations highlight the importance of targeting oxidative stress and mitochondrial damage in ARS-induced nephrotoxicity.

The cellular antioxidant defense system plays a fundamental role in maintaining redox balance and protecting tissues from oxidative injury. SOD converts superoxide radicals into hydrogen peroxide, which is further decomposed by CAT to water and oxygen, thereby limiting hydroxyl radical formation [27]. GPx uses glutathione to detoxify lipid peroxides and hydrogen peroxide, protecting cell membranes and organelles from oxidative damage [28]. The transcription factor NRF2 orchestrates the expression of multiple antioxidant and detoxifying enzymes, with HO-1 as one of its key cytoprotective targets [29,30]. In the current study, ARS significantly increased renal MDA levels, indicating enhanced lipid peroxidation, and reduced the activities of SOD, CAT, and GPx, reflecting an impairment of endogenous antioxidant defenses. These findings agree with reports that ARS suppresses antioxidant enzyme activity while simultaneously elevating ROS production [7]. The suppression of cellular defenses was further supported by decreased expression of NRF2 and HO-1.

Importantly, treatment with MOR, especially in the PLGA nanoparticle formulation, significantly restored SOD, CAT, and GPx activities and enhanced the NRF2/HO-1 pathway, leading to reduced lipid peroxidation and reinforced renal antioxidant defenses. These results are consistent with earlier studies demonstrating the antioxidant and renoprotective effects of MOR in ARS-induced and chemically induced nephrotoxicity [13,31]. Nanoparticle-based delivery likely augments these effects by increasing MOR bioavailability, prolonging systemic exposure, and enhancing renal cellular uptake [16]. Immunohistochemical findings paralleled the molecular data: ARS-treated kidneys showed reduced NRF2 immunoreactivity, whereas MOR, more prominently MOR–PGNPs, significantly restored NRF2 expression. These observations suggest that MOR exerts its protective action, at least in part, by activating NRF2-dependent antioxidant signaling. This is in line with the broader literature, indicating that flavonoids can promote NRF2 nuclear translocation and upregulate cytoprotective genes [32,33].

Alongside oxidative damage, inflammatory mechanisms play a crucial role in ARS-induced renal toxicity [8]. In this study, ARS-exposed rats exhibited marked activation of the TLR4/NF-κB pathway, with significant upregulation of TLR4 and NF-κB and elevated levels of TNF-α, IL-6, and IL-1β in renal tissue. The TLR4/NF-κB axis is a central mediator of inflammatory responses to toxic insults, with NF-κB regulating numerous genes involved in immunity, inflammation, and cellular stress responses [34]. Immunohistochemistry confirmed strong NF-κB expression in kidneys from ARS-treated animals, indicating inflammatory activation, in agreement with previous reports of ARS-induced NF-κB signaling [4,35]. This activation likely contributes to the increased production of pro-inflammatory cytokines and subsequent renal injury.

Morin treatment significantly suppressed NF-κB activation and reduced pro-inflammatory cytokine levels, underscoring its potent anti-inflammatory properties. These findings align with previous studies showing that MOR inhibits NF-κB nuclear translocation and decreases oxidative-stress-driven inflammation [14]. The nanoparticle formulation of MOR produced even greater suppression of NF-κB, as evidenced by markedly reduced immunoreactivity in renal tissues compared with crude MOR. This suggests that improved delivery and tissue targeting via PLGA nanoparticles potentiates the anti-inflammatory efficacy of MOR.

Oxidative stress and inflammation induced by ARS also drive fibrotic signaling, promoting the progression of kidney fibrosis [9]. In this study, ARS significantly increased renal levels of TGF-β and fibronectin, two key mediators of extracellular matrix accumulation and fibrosis [26,36]. The pronounced elevation of KIM-1 further indicated extensive tubular epithelial injury [37]. These observations are consistent with previous studies demonstrating that TGF-β signaling is central to renal fibrogenesis in nephrotoxic models, including ARS-induced kidney injury [9,38]. Treatment with MOR markedly reduced TGF-β, fibronectin, and KIM-1 levels, indicating attenuation of ARS-induced renal fibrogenesis, with MOR–PGNPs exerting an even stronger antifibrotic effect.

Apoptosis represents another key mechanism underlying ARS-induced nephrotoxi-472 city [4,39]. In the present study, ARS exposure significantly upregulated the pro-apoptotic 473 markers caspase-3, caspase-8, and Bax, while downregulating the anti-apoptotic protein 474 Bcl-2. This shift in the balance between pro- and anti-apoptotic regulators suggests activation of both the intrinsic and extrinsic apoptotic pathways [40]. Bax promotes mitochondrial outer membrane permeabilization and cytochrome c release, thereby facilitating caspase activation [41], while caspase-3 serves as a key executioner caspase responsible for cleaving essential cellular substrates during apoptosis [42]. In contrast, Bcl-2 maintains mitochondrial integrity and inhibits apoptosis [43]. Our findings concur with earlier reports that ARS triggers mitochondrial dysfunction and apoptosis in kidney cells [6,9,44].

MOR treatment, particularly with the PLGA nanoparticle formulation, effectively counteracted these apoptotic changes by reducing caspase-3 and caspase-8 expression and increasing Bcl-2 levels. These effects likely reflect the combined antioxidant and anti-inflammatory actions of MOR, which help preserve mitochondrial function and cellular integrity. Although direct studies on MOR in ARS-induced renal apoptosis are limited, its known protective effects in other nephrotoxic models support a role in modulating apoptotic signaling and preventing renal cell loss [13,31].

Collectively, our biochemical, molecular, histopathological, ultrastructural, and immunohistochemical findings indicate that MOR–PGNPs provide potentially enhanced renoprotection compared with crude MOR against ARS-induced kidney injury. The enhanced efficacy of MOR–PGNPs is likely attributable to potentially improved bioavailability, prolonged circulation, and plausibly better renal tissue exposure achieved through PLGA-based nanodelivery as suggested by the physicochemical properties of the formulation, including its particle size of 118 nm, zeta potential of −24 mV, high entrapment efficiency (82.17%), and sustained release profile [16]. It should be noted, however, that formal pharmacokinetic and biodistribution studies were not conducted in the present work; therefore, these claims should be regarded as supported by indirect evidence rather than direct pharmacokinetic confirmation. Furthermore, the mechanistic conclusions of the present study are inferred from convergent biochemical, molecular, and immunohistochemical evidence rather than directly demonstrated through Western blot analysis of phosphorylated signaling intermediates, Nrf2 nuclear translocation assays, or pathway-specific inhibitor studies; future investigations employing such tools will be essential to formally establish the causal roles of the Nrf2/HO-1 and TLR4/NF-κB pathways in mediating the renoprotective effects of MOR–PGNPs.

Several limitations of this study should be acknowledged. First, experiments were conducted exclusively in male Sprague Dawley rats, and whether the observed effects extend to female animals or to other species remains to be determined. Second, the subacute arsenic exposure model (10 mg/kg for 14 days) does not fully replicate the chronic, low-level exposure encountered in real-world human settings, particularly in mining-affected regions. Furthermore, only a single arsenic dose was employed, and neither a formal dose–response evaluation nor a reaction threshold determination was performed. Future studies incorporating multiple arsenic dose levels, spanning environmentally relevant chronic low-dose exposure to sub-acute suprathreshold concentrations, would provide a more complete toxicological characterization and allow identification of the minimum arsenic concentration at which MOR–PGNPs-based renoprotection remains operative. Third, only a single dose of MOR and MOR–PGNPs (100 mg/kg) was evaluated, as this dose was previously reported to be safe and effective in nephroprotective studies; nevertheless, dose–response investigations are still required to determine the minimum effective dose and the optimal therapeutic window, particularly for the nanoparticle formulation, which may retain efficacy at lower doses due to enhanced bioavailability and sustained release properties. Fourth, formal pharmacokinetic studies, including plasma concentration-time profiling, tissue distribution analysis, and ADME characterization, were not performed in the present work. Therefore, claims of improved bioavailability and enhanced renal tissue exposure should be interpreted as supported by indirect physicochemical and pharmacodynamic evidence rather than direct pharmacokinetic confirmation. In addition, the biodistribution of MOR–PGNPs across organs other than the kidney was not assessed. Dedicated pharmacokinetic and biodistribution studies are essential before any translational or clinical application of MOR–PGNPs can be considered. Finally, the mechanistic analysis was confined to a targeted marker panel, assessed by ELISA, RT-PCR, and immunohistochemistry; although convergent evidence across protein, gene, and tissue levels supports pathway engagement, formal mechanistic causality was not established through Western blot analysis of phosphorylated signaling proteins, Nrf2 nuclear translocation assays, or pathway-specific inhibitor studies, all of which are prioritized as future research directions. Broader omics approaches may reveal additional pathways not captured here. Collectively, these limitations highlight important directions for future research and should be considered when interpreting the translational relevance of the current findings.

## 4. Materials and Methods

### 4.1. Preparation of Morin-Loaded PLGA Nanoparticles (MOR–PGNPs)

Morin-loaded PLGA nanoparticles were prepared using a modified nanoprecipitation/emulsification method adapted from a previous study [45]. Briefly, 100 mg of morin and 200 mg of PLGA (50:50 lactide: glycolide) were dissolved in 3 mL of acetone to constitute the organic phase. In parallel, 20 mL of an aqueous poly(vinyl alcohol) (PVA) solution (1–2% *w*/*v*), used as a stabilizer, was cooled in an ice bath to maintain low-temperature conditions during nanoparticle formation.

The organic phase containing morin and PLGA was slowly added dropwise to the chilled PVA solution under continuous high-speed homogenization using an Ultra-Turrax homogenizer at 15,000 rpm for 5 min, yielding a coarse emulsion. To further reduce particle size and obtain a uniform nanosuspension, the emulsion was immediately subjected to probe sonication at 80% amplitude, applying repeated 10 s pulses for an optimized duration. The resulting morin–PLGA nanosuspension was then stirred at room temperature for approximately 3 h on a magnetic stirrer to ensure complete evaporation of the organic solvent and stabilization of the nanoparticles.

The final nanosuspension was collected, transferred to airtight containers, and stored at 4 °C until further characterization and in vivo use. All preparations were performed under aseptic conditions to minimize contamination.

### 4.2. Characterization of MOR–PGNPs

The physicochemical properties of the morin–PLGA nanoparticles were evaluated by measuring hydrodynamic diameter, PDI, and zeta potential using a dynamic light scattering (DLS) instrument equipped with electrophoretic mobility analysis. Samples were appropriately diluted with deionized water prior to analysis to avoid multiple scattering. The Z-average diameter and PDI were recorded to assess size and size distribution, and zeta potential was measured in triplicate to ensure reproducibility and to infer colloidal stability.

Nanoparticle morphology and surface characteristics were examined by transmission electron microscopy (TEM) (Supplementary Table S1). A drop of the diluted nanosuspension was placed on a carbon-coated copper grid, allowed to adsorb for a few minutes, blotted to remove excess fluid, and air-dried. TEM images were acquired at an accelerating voltage of 160 kV, and representative micrographs were used to confirm particle shape and dispersion.

### 4.3. Stability Study

The physical stability of morin-loaded PLGA nanoparticles (MOR–PGNPs) was evaluated by monitoring changes in particle size, PDI, and zeta potential during storage. The freshly prepared nanosuspension was stored in airtight containers at 4 °C for several days. At predetermined time intervals (0, 3, 7, and 14 days), samples were withdrawn and analyzed for their hydrodynamic diameter, PDI, and zeta potential using dynamic light scattering (DLS) under the same measurement conditions described previously. All measurements were performed in triplicate to ensure reproducibility.

### 4.4. Entrapment Efficiency

Entrapment efficiency (EE) and drug loading (DL) of morin in the PLGA nanoparticles were determined indirectly by quantifying the amount of free (non-encapsulated) morin remaining in the supernatant after nanoparticle formation. The nanosuspension was centrifuged, and the concentration of unencapsulated morin in the clear supernatant was measured spectrophotometrically at the appropriate wavelength using a calibration curve prepared from known morin standards.

Entrapment efficiency was calculated using the following equation:

“EE(%) = [(Initial drug amount − Free drug amount)/Initial drug amount] × 100”

Drug loading (DL) was calculated to determine the percentage of morin incorporated within the nanoparticle matrix relative to the total weight of nanoparticles according to the following equation:

“DL(%) = [Amount of encapsulated drug/Total weight of nanoparticles] × 100”

This approach provided an estimate of the fraction of morin successfully incorporated into the nanoparticle matrix.

### 4.5. FTIR Analysis

Fourier-transform infrared (FTIR) spectroscopy (Supplementary Table S1) was employed to investigate potential interactions between morin and PLGA and to confirm successful encapsulation. Samples of pure morin, PLGA, and freeze-dried morin–PLGA nanoparticles were individually mixed with spectroscopic-grade potassium bromide (KBr) and compressed into transparent pellets. FTIR spectra were recorded over the range 4000–400 cm^−1^ at room temperature. Characteristic absorption bands of morin, PLGA, and the nanoformulation were compared to identify shifts or changes indicative of hydrogen bonding or other non-covalent interactions between the drug and polymer.

### 4.6. In Vitro Drug Release Study

The in vitro release profile of morin from PLGA nanoparticles was assessed using a dialysis bag diffusion method. A known volume of the morin-loaded nanosuspension was placed into pre-soaked dialysis tubing and immersed in phosphate-buffered saline (PBS, pH 7.4) maintained at 37 °C under gentle agitation to mimic physiological conditions. At predefined time points over 72 h, aliquots of the external release medium were withdrawn and immediately replaced with equal volumes of fresh PBS to maintain sink conditions.

The concentration of morin in the collected samples was quantified spectrophotometrically using a standard calibration curve, and the cumulative percentage of drug released was calculated over time. This experiment provided information on the sustained-release behavior and release kinetics of morin from the PLGA nanoparticles.

### 4.7. In Vitro Release Kinetic Modeling

To further elucidate the mechanism of morin release from PLGA nanoparticles, the in vitro release data were fitted to different mathematical kinetic models, including zero-order, first-order, Higuchi, and Korsmeyer-Peppas models. The cumulative percentage of drug release versus time data was analyzed by linear regression after appropriate transformation for each model.

For zero-order kinetics, cumulative drug release was plotted as a function of time. First-order kinetics were evaluated by plotting the logarithm of the remaining drug versus time. The Higuchi model was assessed by plotting cumulative drug release versus the square root of time. The Korsmeyer–Peppas model was applied using the log–log plot of cumulative drug release versus time to determine the release exponent (n), which provides insight into the release mechanism.

The best-fit model was selected based on the highest correlation coefficient (R^2^), and the release exponent from the Korsmeyer–Peppas model was used to interpret the drug release mechanism from the PLGA matrix.

The regression analysis for all kinetic models was performed using appropriate software, and the correlation coefficients (R^2^) were compared to identify the most suitable model describing the drug-release behavior.

### 4.8. Experimental Animals and Study Design

Sixty healthy male Sprague Dawley rats (albino strain), weighing 200–250 g, were obtained from the animal facility of MERC, Mansoura University, Egypt. Male rats were selected to avoid the potential influence of hormonal fluctuations associated with the estrous cycle in females, which may affect oxidative stress, inflammatory responses, and renal function parameters, thereby introducing variability in the experimental outcomes. In addition, male rats are commonly used in arsenic-induced nephrotoxicity studies to ensure consistency and comparability with previously published data [46,47].

Upon arrival, animals were examined for general health and housed in clean, ventilated plastic cages (standard group housing) under controlled environmental conditions (temperature 22–25 °C, relative humidity 45–60%, 12 h light/12 h dark cycle). Rats had free access to standard laboratory chow and tap water ad libitum and were allowed a 2-week acclimatization period before experiments to minimize stress-related variability.

All procedures were conducted in accordance with international guidelines for the care and use of laboratory animals and complied with the principles of the OECD 420 guideline for acute oral toxicity and related safety considerations. The study protocol was reviewed and approved by the local Institutional Animal Care and Use Committee (MU-ACUC; VM.R.26.04.282).

Animals were randomly allocated to six experimental groups (n = 10 per group) using a simple randomization procedure. The experimental unit was the individual animal. The groups were as follows:

- Group I (Control): received corn oil orally.
- Group II (MOR): received morin (100 mg/kg) by oral gavage.
- Group III (MOR–PGNPs): received an equivalent dose of morin encapsulated in PLGA nanoparticles (100 mg/kg, morin basis) by oral gavage.
- Group IV (ARS): received arsenic (ARS) orally at 10 mg/kg for 14 consecutive days to induce sub-acute nephrotoxicity, following Azmat et al. [6]. This dose is higher than those encountered in typical environmental exposures and is required to produce reproducible, measurable renal injury within a defined experimental timeframe, reflecting the higher metabolic rate and renal clearance capacity of rats compared with humans [4,6].
- Group V (ARS + MOR): received ARS (10 mg/kg) plus morin (100 mg/kg).
- Group VI (ARS + MOR–PGNPs): received ARS (10 mg/kg) plus MOR–PGNPs (100 mg/kg, morin equivalent).

In the co-treatment groups, MOR or MOR–PGNPs were administered by oral gavage approximately 30 min after the ARS dose to allow initial gastric processing and intestinal absorption of arsenic prior to the protective intervention, consistent with gastric emptying kinetics in rats [48] and the reported gradual absorption profile of orally administered arsenic [49]. The selected MOR dose was based on previous experimental studies demonstrating significant antioxidant, anti-inflammatory, and renoprotective effects at this dose without evidence of toxicity. Due to MOR’s poor oral bioavailability and rapid metabolism, relatively higher doses are often required to achieve effective therapeutic concentrations in target tissues [6,13]. In addition, the MOR–PGNP formulation was characterized for encapsulation efficiency and drug-release behavior to confirm stable incorporation of MOR within the PLGA nanoparticles and minimize premature drug leakage. Of the 10 rats per group, 7 were designated for biochemical and molecular analyses, while the remaining three served as reserves to replace any animals that died or developed health complications during the study period.

### 4.9. Animal Handling and Sample Collection

Animal handling, euthanasia, blood collection, serum preparation, and renal tissue processing procedures were carried out following the same protocol described in detail in our previous publication [50]. Briefly, rats were fasted for approximately 10 h prior to euthanasia, which was performed humanely using inhaled isoflurane. Within each group, seven rats were allocated to biochemical and molecular analyses, and three to histopathological and ultrastructural assessments. Serum was separated by centrifugation and stored at −80 °C, and kidney homogenates were prepared in ice-cold Tris–HCl buffer (50 mmol/L, pH 7.4) and stored at −20 °C until analysis.

### 4.10. Evaluation of Kidney Function

Serum creatinine, urea, and uric acid were determined using standard kinetic or endpoint colorimetric methods, as described [51]. Commercial kits were used according to the manufacturers’ instructions, and absorbance was measured at the recommended wavelengths using a spectrophotometer. Exact kit types, catalog numbers, and suppliers are listed in Supplementary Table S1.

Renal KIM-1 was quantified in kidney tissue homogenates using a sandwich ELISA according to the manufacturer’s protocol. Briefly, samples and standards were added to pre-coated wells, followed by incubation with specific detection antibodies and horseradish peroxidase (HRP)-conjugated streptavidin. After washing, color was developed with chromogenic substrate, and absorbance was read at 450 nm. KIM-1 concentrations were interpolated from a standard curve and expressed relative to tissue protein content.

### 4.11. Evaluation of Antioxidant Defenses and Oxidative Stress Biomarkers

Activities of the antioxidant enzymes SOD, CAT, and GPx in kidney homogenates were determined using validated colorimetric assays, according to the instructions provided with the commercial kits and as previously demonstrated [52,53,54]. Renal Nrf2 and HO-1 protein levels were assessed using rat-specific ELISA kits, and concentrations were calculated from standard curves. Lipid peroxidation was evaluated by determining malondialdehyde (MDA) levels (per milligram of tissue protein). Details of all kits and suppliers are provided in Supplementary Table S1.

### 4.12. Intracellular Reactive Oxygen Species (ROS)

Intracellular ROS levels in kidney cells were assessed using a fluorescence-based assay employing a cell-permeable probe (e.g., DCFH-DA). Fresh kidney tissue was gently dissociated to obtain single-cell suspensions, which were incubated with the fluorescent probe at 37 °C for 30 min in the dark. After washing to remove excess dye, fluorescence intensity was measured with a microplate reader at appropriate excitation/emission wavelengths (approximately 488/525 nm). ROS levels were expressed as relative fluorescence units (RFU) and normalized to control values.

### 4.13. Evaluation of Renal Inflammation Biomarkers

Renal inflammation was characterized by measuring NF-κB, TNF-α, IL-1β, IL-6, and TLR4 in kidney homogenates using rat-specific ELISA kits. Samples and standards were added to antibody-coated plates and incubated with target-specific detection antibodies and HRP-conjugated streptavidin according to the manufacturer’s protocols. After washing, color was developed with substrate, the reaction was stopped, and absorbance was read at 450 nm. Cytokine and TLR4 concentrations were calculated from standard curves and normalized to tissue protein content. Kit details and suppliers are summarized in Supplementary Table S1.

### 4.14. Assessment of Renal Fibrosis Biomarkers

Markers of renal fibrosis were evaluated by quantifying TGF-β and fibronectin in kidney tissue homogenates using ELISA-based assays. In brief, samples and standards were added to wells pre-coated with specific capture antibodies, incubated with detection antibodies and an HRP-conjugate, and developed with a chromogenic substrate. Absorbance was measured at 450 nm, and concentrations were derived from standard curves. Results were expressed relative to total protein content to allow comparison across groups.

### 4.15. Assessment of Renal Apoptosis Biomarkers

Renal apoptosis was assessed by determining caspase-3 and caspase-8 activity using fluorescence-based assay kits (Caspase-3 Fluorometric Assay Kit, Catalog No. K105, BioVision Inc., Milpitas, CA, USA; and Caspase-8 Fluorometric Assay Kit, Catalog No. K113, BioVision Inc., Milpitas, CA, USA). Kidney homogenates were incubated with specific peptide substrates labeled with fluorescent reporters (DEVD-AFC for caspase-3 and IETD-AFC for caspase-8). The release of the fluorophore upon enzymatic cleavage was monitored at excitation/emission wavelengths of 400/505 nm. Enzyme activities were normalized to total protein concentration.

### 4.16. RNA Extraction and Quantitative RT-PCR Analysis

Total RNA was extracted from kidney tissue using a monophasic QIAzol or similar phenol-guanidinium-based lysis reagent (Supplementary Table S1). Briefly, tissue samples were homogenized thoroughly in lysis reagent using a bead-based tissue disruptor to maximize RNA yield. After the addition of chloroform, the samples were vigorously mixed and centrifuged at 12,000× *g* for 15 min to separate the phases. The aqueous phase containing RNA was carefully collected, and RNA was precipitated with isopropanol and centrifuged at 12,000× *g* for 10 min. The resulting pellet was washed with ethanol, centrifuged again to remove residual ethanol, air-dried, and dissolved in DNase/RNase-free water. RNA concentration and purity were assessed spectrophotometrically, and integrity was checked before downstream applications.

Complementary DNA (cDNA) was synthesized from total RNA using a commercially available reverse transcription kit, following the manufacturer’s instructions (Supplementary Table S1). Quantitative real-time PCR (qPCR) was performed using a SYBR Green-based master mix on a Rotor-Gene Q or equivalent real-time PCR platform. Gene-specific primers (listed in Table 2) were used to amplify targets related to antioxidant defenses, inflammation, fibrosis, and apoptosis, along with an appropriate housekeeping gene, tested for its stability [55].

Amplification conditions were optimized for each primer pair. Relative gene expression was calculated using the 2^−ΔΔCt^ method described by Livak and Schmittgen [56], with the control group serving as the calibrator. All reactions were run in at least technical duplicates, and no-template controls were included to check for contamination.

### 4.17. Histopathological Analysis

Tissue fixation, paraffin embedding, sectioning, and hematoxylin and eosin (H&E) staining were performed according to the detailed protocol described in our previous publication [50]. Briefly, kidney specimens were fixed in 10% neutral-buffered formalin, processed through graded ethanol and xylene, embedded in paraffin, and sectioned using a rotary microtome. For each experimental group, three rats were evaluated; three sections per rat were prepared, and four non-overlapping fields per section were examined at 400× magnification, yielding 12 fields per animal. Renal tissue damage was scored semi-quantitatively from 0 (absent) to 3 (severe) across four parameters, tubular damage/necrosis, glomerular structural alterations, inflammatory cell infiltration, and hemorrhage, using the predefined AASLD-based criteria detailed in Table 3. The mean score across all evaluated fields was calculated per animal.

### 4.18. Transmission Electron Microscopy (TEM)

For ultrastructural analysis, small kidney tissue fragments were fixed in 2.5% glutaraldehyde prepared in 0.1 M phosphate buffer (pH 7.2–7.4) and stored at 4 °C for approximately 24 h. Specimens were then washed several times in phosphate buffer to remove residual fixative and post-fixed in 1% osmium tetroxide for 2 h to enhance membrane preservation and contrast.

After post-fixation, tissues were dehydrated through a graded ethanol series (50–100%) and then treated with acetone prior to embedding in epoxy resin. Ultrathin sections (approximately 60–70 nm) were cut using an ultramicrotome and mounted on copper grids. Sections were stained sequentially with uranyl acetate and lead citrate to enhance visualization of cellular and subcellular structures. Ultrastructural changes in renal tissue were examined and photographed using a JEOL JEM-2100 transmission electron microscope (JEOL Ltd., Tokyo, Japan) operated at 160 kV (Supplementary Table S1).

### 4.19. Immunohistochemical Detection of Nrf2 and NF-κB

Paraffin-embedded kidney sections were used for immunohistochemical detection of Nrf2 and NF-κB. Sections were deparaffinized in xylene, rehydrated through graded ethanols (100% to 70%), and rinsed in distilled water. Antigen retrieval was performed by heating sections in 0.01 M citrate buffer (pH 6.0) in a microwave oven for approximately 10 min. Endogenous peroxidase activity was blocked by incubating sections in 3% hydrogen peroxide in methanol.

To minimize nonspecific binding, sections were incubated with 5% bovine serum albumin (BSA) for 30 min, followed by overnight incubation at 4 °C with the following primary antibodies: anti-Nrf2 rabbit recombinant monoclonal antibody (clone EP1808Y, ab62352, Abcam, Cambridge, UK) at a dilution of 1:200, and anti-NF-κB p65 rabbit recombinant monoclonal antibody (clone E379, ab32536, Abcam, Cambridge, UK) at a dilution of 1:200. After rinsing in PBS, sections were incubated with biotinylated goat anti-rabbit secondary antibody (ab6720, Abcam) at a 1:500 dilution for 30 min at room temperature, followed by streptavidin–HRP conjugate (ab7403, Abcam). Signal was visualized using a 3,3′-diaminobenzidine (DAB) chromogen substrate kit (ab64238, Abcam), yielding brown staining in antigen-positive cells. Slides were counterstained with Mayer’s hematoxylin, dehydrated, cleared, and mounted.

For each section, five random cortical fields were examined at 400× magnification using a Leica DM750 light microscope (Leica Biosystem, Nussloch, Germany), and digital images were captured under identical exposure settings. Positive immunostaining was quantified using ImageJ software (version 1.54, NIH, Bethesda, MD, USA) by measuring the mean optical density (MOD) of DAB-stained areas after color deconvolution to isolate the DAB channel from the hematoxylin counterstain. The percentage of positively stained area, relative to the total field area was calculated for each image, and the mean value across all five fields per section was recorded as the staining intensity score for each animal. Results were expressed as mean ± SE across all animals per group and compared statistically using one-way ANOVA with Tukey’s HSD post hoc test. Exact antibody clones, dilutions, and suppliers are listed in Supplementary Table S1.

### 4.20. Statistical Analysis

Data were first assessed for normality and homogeneity of variance using the Shapiro–Wilk and Levene’s tests, respectively. For normally distributed data with equal variances, one-way analysis of variance (ANOVA) was performed to compare experimental groups. When the ANOVA indicated significant differences, Tukey’s honestly significant difference (HSD) post hoc test was applied for multiple comparisons.

Results are presented as mean ± standard error (SE). A *p*-value < 0.05 was considered statistically significant. Statistical analyses were conducted using SAS software 9.4 (Proc ANOVA), and graphs were generated with GraphPad Prism version 9.1.0 (GraphPad Software, USA) (Supplementary Table S1). The experimental unit was the individual animal; no data were excluded unless predefined quality criteria were not met, and analysts were blinded to group allocation during histopathological and immunohistochemical scoring whenever feasible.

## 5. Conclusions

Morin-loaded PLGA nanoparticles (MOR–PGNPs) effectively protected rats from arsenic-induced kidney injury. They restored kidney function markers, enhanced antioxidant defenses, reduced oxidative stress, and suppressed inflammatory responses. MOR–PGNPs also attenuated fibrosis and apoptosis, preserving normal renal histology and ultrastructure. Compared with crude MOR, the nanoparticle formulation exhibited likely improved bioavailability and therapeutic efficacy. Taken together, these findings highlight PLGA-based MOR nanoparticles as a promising renoprotective strategy for preventing or mitigating arsenic-induced nephrotoxicity. However, a comprehensive long-term safety evaluation, including biodistribution and chronic toxicity studies, is warranted before clinical translation. These results support further investigation of MOR–PGNPs as a potential therapeutic approach in oxidative stress-mediated renal disorders.

## Figures and Tables

**Table 1:** Effect of storage at 4 °C on the physicochemical stability of MOR–PGNPs over 14 days.

Storage Time (Days)	Particle Size (nm)	PDI	Zeta Potential (mV)
0	118 ± 2.33	0.403 ± 0.02	−24.0 ± 0.76
3	119 ± 3.21	0.406 ± 0.01	−23.1 ± 0.74
7	121 ± 3.08	0.407 ± 0.02	−22.4 ± 0.69
14	125 ± 2.35	0.411 ± 0.03	−21.2 ± 0.91

PDI: Polydispersity index. Data are expressed as mean ± SD.

**Table 2:** Sequences of primers used to evaluate gene expression in kidney tissue.

Gene	Sense (5′-3′)	Antisense (5′-3′)
*Nrf2*	F: TTTGTAGATGACCATGAGTC	R: TCCTGCCAAACTTGCTCCAT
*HO-1*	F: ATGTCCCAGGATTTGTCCGA	R: ATGGTACAAGGAGGCCATCA
*CAT*	F: AGCGACCAGATGAAGCAGTG	R: TCCGCTCTCTGTCAAAGTGT
*SOD*	F: AACCAGTTGTGTTGTCAGG	R: CCACCATGTTTCTTAGAGTGA
*GPX1*	F: AGTTCGGACATCAGGAGAATGGCA	R: TCACCATTCACCTCGCACTTCTCA
*NFκB*	F: AGTCCCGCCCCTTCTAAAAC	R: CAATGGCCTCTGTGTAGCCC
*TLR4*	F: ATCATCCAGGAAGGCTTCCA	R: GCTGCCTCAGCAAGGACTTC
*TNF-α*	F: CTCGAGTGACAAGCCCGTAG	R: ATCTGCTGGTACCACCAGTT
*IL-6*	F: AGCGATGATGCACTGTCAGA	R: GGAACTCCAGAAGACCAGAGC
*IL-1β*	F: ATGGCAACTGTCCCTGAACT	R: AGTGACACTGCCTTCCTGAA
*Caspase-3*	F: ACTGGAATGTCAGCTCGCAA	R: GCAGTAGTCGCCTCTGAAGA
*Caspase-8*	F: CTGGGAAGGATCGACGATTA	R: CATGTCCTGCATTTTGATGG
*Bax*	F: TTTCATCCAGGATCGAGCAG	R: AATCATCCTCTGCAGCTCCA
*Bcl-2*	F: GACTTTGCAGAGATGTCCAG	R: TCAGGTACTCAGTCATCCAC
*β-Actin*	F: CAGCCTTCCTTCTTGGGTATG	R: AGCTCAGTAACAGTCCGCCT

*Nrf2*: Nuclear factor erythroid 2–related factor 2, *HO-1*: Heme oxygenase-1, *CAT*: catalase, *SOD*: superoxide dismutase, *GPX1*: glutathione peroxidase, *NF-κB*: Nuclear factor kappa-light-chain-enhancer of activated B cells, *TLR4*: Toll-like receptor 4, *TNF-α*: tumor necrosis factor-α, *IL-6*: interleukin-6, *IL-1β*: interleukin-1β, *Caspase 3*: Cysteine-aspartic acid protease 3, *Caspase 8*: Cysteine-aspartic acid protease 8, *Bax*: Bcl-2-associated X protein, *Bcl-2*: B-cell lymphoma 2, *β-Actin*: Beta actin.

**Table 3:** Semi-quantitative histopathological scoring of renal tissue alterations.

Score	Tubular Damage and Necrosis in Kidney Sections	Structural Alterations of Glomeruli	Inflammation	Hemorrhage
0	None	None	None	None
1	Of the 12 kidney fields evaluated, only 1 or 2 showed focal degeneration.	Among the kidney sections evaluated, 1 or 2 exhibited mild focal glomerular atrophy.	Among the 12 renal fields evaluated, just 1 or 2 showed minor infiltration by inflammatory cells.	Among the kidney sections evaluated, 1 or 2 showed mild focal congestion, with occasional hemorrhagic spots.
2	Among the 12 kidney fields evaluated, 3–6 showed degenerative changes characterized by focal epithelial sloughing.	Among the 12 kidney fields evaluated, 3–4 showed mild to moderate glomerular shrinkage.	Among the analyzed kidney sections, 3–4 showed moderate focal infiltration by inflammatory cells.	Among the analyzed kidney sections, 3–4 showed focal hemorrhage along with mild interstitial congestion.
3	Among the 12 kidney fields evaluated, 7–9 displayed severe tubular necrosis.	Among the 12 kidney fields evaluated, 5–6 displayed lamellar fusion.	Among the 12 kidney fields evaluated, 5–7 exhibited notable inflammation.	Among the 12 renal fields evaluated, 5–6 showed marked interstitial congestion and focal hemorrhage.

## Data Availability

The original contributions presented in this study are included in the article/Supplementary Materials. Further inquiries can be directed to the corresponding author.

## Supplementary Materials

The following supporting information can be downloaded at: https://www.mdpi.com/article/10.3390/ph19060871/s1, Table S1: Summary of Kits and reagents used in the present study.

## Abbreviations

The following abbreviations are used in this manuscript:

ARS	Arsenic
MOR	Morin
PLGA	Poly(lactic-co-glycolic acid)
MOR–PGNPs	Morin-loaded PLGA nanoparticles
KIM-1	Kidney injury molecule-1
ROS	Reactive oxygen species
MDA	Malondialdehyde
Nrf2	Nuclear factor erythroid 2-related factor 2
HO-1	Heme oxygenase-1
CAT	Catalase
SOD	Superoxide dismutase
GPx	Glutathione peroxidase
TLR4	Toll-like receptor 4
NF-κB	Nuclear factor kappa-B
TNF-α	Tumor necrosis factor-alpha
IL-6	Interleukin-6
IL-1β	Interleukin-1 beta
TGF-β1	Transforming growth factor-beta 1
Bax	Bcl-2-associated X protein
Bcl-2	B-cell lymphoma 2
FTIR	Fourier transform infrared spectroscopy
TEM	Transmission electron microscopy
EE	Entrapment efficiency
DLS	Dynamic light scattering
PDI	Polydispersity index
PBS	Phosphate-buffered saline
BSA	Bovine serum albumin
DAB	3,3′-Diaminobenzidine
ANOVA	Analysis of variance
HSD	Honestly significant difference
SE	Standard error
