# Supplementary material for: Enhanced Renoprotective Effects of Morin-Loaded PLGA Nanoparticles Against Arsenic-Induced Kidney Injury in Rats: Amelioration of Oxidative Stress, Inflammation, Fibrosis, and Apoptosis
Source: Pharmaceuticals (Basel). 2026 May 30;19(6):871. doi: 10.3390/ph19060871 (PMC13306147; doi:10.3390/ph19060871)
Supplement: Supplementary file 1 [file pharmaceuticals-19-00871-s001.zip › pharmaceuticals-4295658-supplementary.pdf]

Reagent/Instrument	Category	Purpose/Use	Manufacturer/Supplier	Country	Catalog/Model
PLGA (50:50)	Polymer	Nanoparticle formulation	Sigma-Aldrich (MilliporeSigma)	MO, USA	719870 (Resomer® RG 503 H; lactide:glycolide 50:50; Mw 24,000–38,000; acid terminated)
Morin	Compound	Active compound for nanoparticle loading and treatment	Sigma-Aldrich (MilliporeSigma)	MO, USA	M4008 (Morin hydrate; ≥85% by HPLC; CAS 654055-01-3)
Poly(vinyl alcohol) (PVA)	Stabilizer	Stabilizer during nanoparticle preparation	Sigma-Aldrich (MilliporeSigma)	MO, USA	363170 (Mw 13,000–23,000; 87–89% hydrolyzed
IKA Ultra-Turrax T25 homogenizer	Instrument	Homogenization during nanoparticle preparation	IKA-Werke GmbH & Co. KG	Staufen, Germany	Ultra-Turrax T25
Bandelin Sonopuls HD 3200	Instrument	Probe sonication during nanoparticle preparation	Bandelin Electronic GmbH & Co. KG	Berlin, Germany	HD 3200
IKA C-MAG HS 7 magnetic stirrer	Instrument	Solvent evaporation and nanosuspension stabilization	IKA-Werke GmbH & Co. KG	Staufen, Germany	C-MAG HS 7
Zetasizer NanoZS	Instrument	Particle size, PDI, and zeta potential measurement	Malvern Panalytical (formerly Malvern Instruments)	Malvern, Worcestershire, UK	ZEN3600
JEOL JEM-2100 / JEOL 2100	Instrument	Transmission electron microscopy for nanoparticle and kidney ultrastructure analysis	JEOL	Japan	JEM-2100 / 2100
Potassium bromide (KBr)	Reagent	Pellet preparation for FTIR analysis	Sigma-Aldrich (MilliporeSigma)	MO, USA	221864 (FT-IR grade, ≥99%)
FTIR spectrometer	Instrument	FTIR analysis of morin, PLGA, and MOR-PGNPs	PerkinElmer	Waltham, MA, USA	Spectrum Two
Phosphate-buffered saline (PBS), pH 7.4	Buffer	Release medium in in vitro drug release assay	Sigma-Aldrich (MilliporeSigma)	MO, USA	P5119 (powder, pH 7.4)
Creatinine assay kit	Kit	Serum creatinine measurement	BioDiagnostic	Giza, Egypt	CR 12 51
Urea assay kit	Kit	Serum urea measurement	BioDiagnostic	Giza, Egypt	UR 21 10
Uric acid assay kit	Kit	Serum uric acid measurement	BioDiagnostic	Giza, Egypt	UA 21 20
Rat KIM-1 ELISA	Kit	Renal KIM-1 quantification	MyBioSource	San Diego, CA, USA	MBS762913
SOD assay	Kit	Renal superoxide dismutase activity	BioDiagnostic	Giza, Egypt	SD 25 21
CAT assay	Kit	Renal catalase activity	BioDiagnostic	Giza, Egypt	CA 25 17
GPx assay	Kit	Renal glutathione peroxidase activity	BioDiagnostic	Giza, Egypt	GP 25 24
Rat Nrf2 ELISA	Kit	Renal Nrf2 quantification	MyBioSource	San Diego, CA, USA	MBS3807961
Rat HO-1 ELISA	Kit	Renal HO-1 quantification	MyBioSource	San Diego, CA, USA	MBS2024438
TBARS/MDA assay	Kit	Assessment of renal lipid peroxidation	BioDiagnostic	Giza, Egypt	MD 2529
Intracellular ROS fluorescent assay	Kit	Measurement of renal intracellular ROS	MyBioSource	San Diego, CA, USA	MBS2540517
Rat NF-kB ELISA	Kit	Renal NF-kB quantification	MyBioSource	San Diego, CA, USA	MBS287521
Rat TNF-a ELISA	Kit	Renal TNF-a quantification	MyBioSource	San Diego, CA, USA	MBS282960
Rat IL-1b ELISA	Kit	Renal IL-1b quantification	MyBioSource	San Diego, CA, USA	MBS232385
Rat IL-6 ELISA	Kit	Renal IL-6 quantification	MyBioSource	San Diego, CA, USA	MBS2885203
Rat TLR4 ELISA	Kit	Renal TLR4 quantification	MyBioSource	San Diego, CA, USA	MBS161614

Rat TGF-b ELISA	Kit	Renal TGF-b quantification	MyBioSource	San Diego, CA, USA	MBS160117
Rat fibronectin ELISA	Kit	Renal fibronectin quantification	MyBioSource	San Diego, CA, USA	MBS761397
Apo-Alert caspase-3 assay	Kit	Assessment of renal caspase-3 activity	Takara Bio USA	Mountain View, CA, USA	630215
Apo-Alert caspase-8 assay kit	Kit	Assessment of renal caspase-8 activity	Takara Bio USA	Mountain View, CA, USA	630211
QIAzol lysis reagent	Reagent	Phenol/guanidinium reagent for total RNA extraction from kidney tissue	QIAGEN GmbH	Hilden, Germany	79306
TissueLyser II	Instrument	Kidney tissue homogenization for RNA extraction	QIAGEN GmbH	Hilden, Germany	TissueLyser II
iScript cDNA Synthesis Kit	Kit	cDNA synthesis	Bio-Rad Laboratories	Hercules, CA, USA	1708891
iTaq Universal SYBR Green Supermix	Reagent	qPCR amplification	Bio-Rad Laboratories	Hercules, CA, USA	1725121
Rotor-Gene Q	Instrument	Quantitative real-time PCR	QIAGEN GmbH	Hilden, Germany	9001861
Rotary microtome	Instrument	Paraffin sectioning for histopathology	Leica Biosystems	Nussloch, Germany	Leica RM2235
Leica DM750 light microscope	Instrument	Histopathological and immunohistochemical evaluation	Leica Microsystems	Wetzlar, Germany	—
Hematoxylin and eosin (H&E)	Stain	Routine histopathological staining	Sigma-Aldrich	MO, USA	Hematoxylin: H9627; Eosin Y: E4382
Glutaraldehyde (EM-grade)	Fixative	Primary fixation for TEM	Electron Microscopy Sciences	Hatfield, PA, USA	16020 (25% glutaraldehyde EM-grade stock, diluted to 2.5%)
Osmium tetroxide (OsO_4)	Fixative	Post-fixation for TEM	Electron Microscopy Sciences	Hatfield, PA, USA	19150 (4% aqueous OsO_4 stock, diluted to 1%)
Epoxy Resin	Embedding kit	Embedding medium	Electron Microscopy Sciences	Hatfield, PA, USA	14120
Uranyl acetate	Stain	TEM contrast staining	Electron Microscopy Sciences	Hatfield, PA, USA	22400 (uranyl acetate, 2% aqueous)
Lead citrate	Stain	TEM contrast staining	Electron Microscopy Sciences	Hatfield, PA, USA	17800 (lead citrate stain, Reynold's formula)
Ultramicrotome	Instrument	Preparation of ultrathin sections (60–70 nm) from epoxy resin-embedded kidney tissue	Leica Biosystems	Nussloch, Germany	Leica EM UC7
Transmission electron microscope (TEM)	Instrument	Ultrastructural examination and imaging of renal tissue to evaluate subcellular changes	JEOL Ltd.	Tokyo, Japan	JEOL JEM-2100
Anti-Nrf2 primary antibody [EP1808Y]-rabbit recombinant monoclonal	Antibody	Immunohistochemical detection of Nrf2	Abcam	Cambridge, United Kingdom	ab62352
Anti-NF-kB p65 primary antibody [E379]-rabbit recombinant monoclonal	Antibody	Immunohistochemical detection of NF-kB p65	Abcam	Cambridge, United Kingdom	ab32536
Biotinylated goat anti-rabbit IgG H&L (Biotin)	Antibody	Secondary antibody for IHC	Abcam	Cambridge, United Kingdom	ab6720 (concentrated stock, compatible with 1:500 dilution for IHC)
Bovine serum albumin (BSA)	Blocking reagent	Blocking nonspecific binding in IHC	Sigma-Aldrich	MO, USA	A3294 (heat shock fraction, ≥98%, protease-free)

Citrate buffer (0.01 M, pH 6.0)	Buffer	Antigen retrieval in IHC	Sigma-Aldrich	MO, USA	C9999 (10× concentrate, diluted to working concentration)
Hydrogen peroxide in methanol	Reagent	Blocking endogenous peroxidase in IHC	Sigma-Aldrich	MO, USA	H1009 (30% H_2O_2 stock, diluted to 3% in methanol)
Streptavidin-HRP	Detection reagent	Signal development in ELISA/IHC	Abcam	Cambridge, United Kingdom	ab7403
DAB chromogen	Detection reagent	Visualization of IHC positive staining	Abcam	Cambridge, United Kingdom	ab64238 (DAB Substrate Kit)
Mayer's hematoxylin	Counterstain	Counterstaining in IHC	Sigma-Aldrich	MO, USA	MHS32
ImageJ	Software	Quantification of positive immunostaining	National Institutes of Health (NIH/open-source), Bethesda, MD, USA; https://imagej.nih.gov)	Bethesda, MD, USA	ImageJ v. 1.54 (https://imagej.nih.gov)
Statistical Analysis System (SAS)	Software	Statistical analysis with PROC ANOVA for intergroup comparisons	SAS Institute	Cary, NC, USA	SAS 9.4
GraphPad Prism	Software	Graph preparation and visualization	GraphPad Software	San Diego, CA, USA	version 9.1.0, build 221
